# Reading and Modulating Cortical β Bursts from Motor Unit Spiking Activity

**DOI:** 10.1523/JNEUROSCI.1885-21.2022

**Published:** 2022-04-27

**Authors:** Mario Bräcklein, Deren Y. Barsakcioglu, Alessandro Del Vecchio, Jaime Ibáñez, Dario Farina

**Affiliations:** ^1^Neuromechanics and Rehabilitation Technology Group, Department of Bioengineering, Faculty of Engineering, Imperial College London, London W12 0BZ, United Kingdom; ^2^Department Artificial Intelligence in Biomedical Engineering, Friedrich-Alexander University Erlangen-Nuremberg, Erlangen 91052, Germany; ^3^Biomedical Signal Interpretation and Computational Simulation (BSICoS), Instituto de Investigación Sanitaria Aragón, Universidad de Zaragoza, Zaragoza 50018, Spain; ^4^Department of Clinical and Movement Disorders, Institute of Neurology, University College London, London WC1N 3BG, United Kingdom

**Keywords:** β oscillations, motor units, neural interfaces, neural oscillations, real-time decomposition

## Abstract

β Oscillations (13–30 Hz) are ubiquitous in the human motor nervous system. Yet, their origins and roles are unknown. Traditionally, β activity has been treated as a stationary signal. However, recent studies observed that cortical β occurs in “bursting events,” which are transmitted to muscles. This short-lived nature of β events makes it possible to study the main mechanism of β activity found in the muscles in relation to cortical β. Here, we assessed whether muscle β activity mainly results from cortical projections. We ran two experiments in healthy humans of both sexes (*N* = 15 and *N* = 13, respectively) to characterize β activity at the cortical and motor unit (MU) levels during isometric contractions of the tibialis anterior muscle. We found that β rhythms observed at the cortical and MU levels are indeed in bursts. These bursts appeared to be time-locked and had comparable average durations (40–80 ms) and rates (approximately three to four bursts per second). To further confirm that cortical and MU β have the same source, we used a novel operant conditioning framework to allow subjects to volitionally modulate MU β. We showed that volitional modulation of β activity at the MU level was possible with minimal subject learning and was paralleled by similar changes in cortical β activity. These results support the hypothesis that MU β mainly results from cortical projections. Moreover, they demonstrate the possibility to decode cortical β activity from MU recordings, with a potential translation to future neural interfaces that use peripheral information to identify and modulate activity in the central nervous system.

**SIGNIFICANCE STATEMENT** We show for the first time that β activity in motor unit (MU) populations occurs in bursting events. These bursts observed in the output of the spinal cord appear to be time-locked and share similar characteristics of β activity at the cortical level, such as the duration and rate at which they occur. Moreover, when subjects were exposed to a novel operant conditioning paradigm and modulated MU β activity, cortical β activity changed in a similar way as peripheral β. These results provide evidence for a strong correspondence between cortical and peripheral β activity, demonstrating the cortical origin of peripheral β and opening the pathway for a new generation of neural interfaces.

## Introduction

Neural oscillations of brain activity in the β range (13–30 Hz) are ubiquitous in the motor nervous system (Kilavik et al., 2013). Alongside their pervasive appearance in the brain, β oscillations with cortical origin are transmitted linearly and at fast and stable speeds to tonically active muscles (Witham et al., 2011; Ibáñez et al., 2021). β Activity can indeed represent an important portion of the neural inputs received by spinal motor neurons and their innervated muscle fibers, i.e., motor units (MUs; Grosse et al., 2002; Farina et al., 2014; Dideriksen et al., 2018). However, the prominence of β activity at the MU level contrasts with the fact that, so far, it has been difficult to find a direct link between these oscillations and motor function (Baker, 2007; Engel and Fries, 2010; Jenkinson and Brown, 2011; Davis et al., 2012; Little et al., 2019). One aspect of β inputs to MU that makes them hard to study is not knowing which main sources are contributing to these inputs. Are the characteristics of β activity in MUs similar to the nonstationary features of β oscillations at the cortical level? Is the motor cortex the main structure projecting common β inputs to muscles? Or are there other relevant sources elsewhere in the central nervous system?

An interesting recent observation is that cortical β activity is not a continuous signal, but it appears in short-lived bursts (Pfurtscheller et al., 2005; Feingold et al., 2015; Shin et al., 2017; Little et al., 2019; Bonaiuto et al., 2021). Such temporal nonstationary characteristics of β activity require new approaches, based on joint time and frequency analysis, to study these oscillations (Jones, 2016; van Ede et al., 2018; Tal et al., 2020) and their possible links to motor function (Shin et al., 2017; Little et al., 2019; Wessel, 2020; Bonaiuto et al., 2021). The tracking of the nonstationary, burst-like behavior of cortical β allows for directly following its propagation to the peripheral nervous system by identifying its main characteristics, such as burst duration and frequency, at the cortical and peripheral level. The analysis of the transmission of β from the central to the peripheral nervous system would provide new insights into the role of β oscillations on motor control. Moreover, understanding β transmission would enable the development of neural interfaces to monitor and extract cortical activity noninvasively from the periphery to supplement and overcome current limitations of traditional brain monitoring interfaces.

Here, we ran two experiments to characterize β oscillations present at the level of MUs in the tibialis anterior muscle and their association with cortical β rhythms in the context of mild isometric contractions. In the first experiment, we asked subjects to hold a constant force level while concurrently recording cortical activity via electroencephalography (EEG) and muscle activity via high-density electromyography (EMG). The EMG was decomposed into the underlying MU activity associated with force generation. Then, in the second experiment, we used a decomposition algorithm to extract MU activity from the EMG in real time (Barsakcioglu et al., 2021) and a novel neural feedback paradigm to operantly conditioning β in the MUs (Bräcklein et al., 2021). By doing this, we were able to assess how the relationship between cortical and peripheral β rhythms is influenced by volitional modulation of MU β power. Overall, our results demonstrate that β activity in the MUs is short-lived, mainly driven by cortical bursts, and can be volitionally modulated, imposing parallel modulation at the cortical level.

## Materials and Methods

### Subjects

In this study, 28 healthy subjects (three females, all subjects between 24 and 35 years old) participated, of whom 15 (two females) in experiment 1 and 13 (one female) in experiment 2. All subjects were naive to the experimental paradigms. None of the subjects reported any history of sever neuronal or lower limb injuries. Experiment 1 was approved by the University College London Ethics Committee (Ethics Application 10 037/001) and experiment 2 by the ethics committee at Imperial College London (reference number 18IC4685).

### Data acquisition

High-density surface EMG (HDsEMG) from the tibialis anterior muscle of the dominant leg (self-reported) was acquired via a 64-electrode grid (five columns and 13 rows; gold-coated; 1-mm diameter; 8-mm interelectrode distance; OT Bioelettronica). The electrode grid was placed over the muscle belly aligned to the muscle's fiber direction. In addition, single-channel EMG of the medial and lateral head of the gastrocnemius muscle was recorded via wet electrodes (Ambu Ltd) placed above the muscle belly throughout experiment 2. The EMG signals were monopolar recorded, amplified via a Quattrocento Amplifier system (OT Bioelettronica), sampled at 2048 Hz, A/D converted to 16 bits, and digitally bandpass filtered (10–500 Hz). Subjects were seated throughout the experiments while the foot of the dominant leg was locked into position to allow dorsiflexion of the ankle only. The force because of ankle dorsiflexion was recorded via a CCT TF-022 force transducer, amplified (OT Bioelettronica), and low-pass filtered at 33 Hz. The communication between the amplifier and the computer was conducted via data packages of 256 samples (one buffer corresponds to a signal length of 125 ms). All incoming EMG signals were bandpass filtered between 20 and 500 Hz using a fourth order Butterworth filter. Furthermore, EEG signals were acquired from 31 positions according to the International 10–20 system via active Ag/AgCl electrodes (actiCAP, Brain Products GmbH). FCz was used as a reference. The signal was amplified (BrainVision actiCHamp Plus, Brain Products GmbH) and sampled at 1000 Hz. The EEG was offline bandpass filtered between 0.5 and 45 Hz (fourth order Butterworth filter). A surface Laplacian filter covering the central part of the brain by taking the neighboring positions of Cz into account was applied (Kayser and Tenke, 2015). Both EMG and EEG signals were offline resampled at 512 Hz and synchronized with a common digital trigger signal.

For one subject, no EMG of the lateral nor medial head of the gastrocnemius muscle was recorded because of a material failure.

### Experimental paradigm

**Figure 1.**
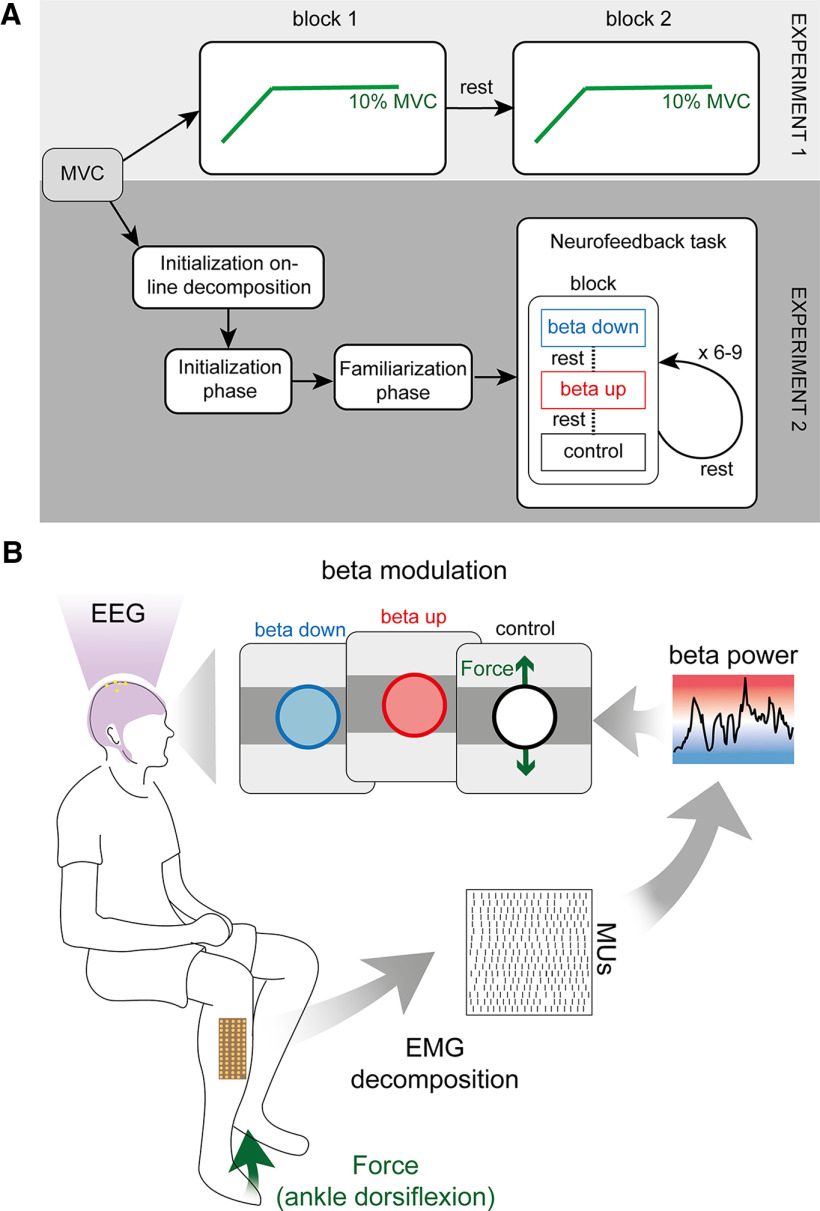
Schematic overview of the experimental paradigms used in both experiments. ***A***, Experimental flowchart for experiments 1 and 2. Both experiments start with estimating the MVC. In experiment 1, subjects are asked to repeat two blocks of ramp-and-hold force task at 10% MVC separated by a rest period. Experiment 2 continues with two initialization steps in which the online decomposition (“Initialization online decomposition”) and the neurofeedback parameters (“Initialization phase”) are initialized. In “Familiarization phase,” subjects are exposed to the neurofeedback paradigm used during the “Neurofeedback task.” A single block of the “Neurofeedback task” consisted of three trails: β down, β up, and control. The trials were presented in randomized order and separated by a rest period. A minimum of six and a maximum of nine blocks were presented to each subject separated by a rest period while only the last three blocks were used for the analysis. ***B***, Schematic overview of experiment 2. HDsEMG of the tibialis anterior muscle was decomposed into the underlying neural activity while, concurrently, the force because of ankle dorsiflexion and the EEG were recorded. Subjects were asked to navigate a cursor inside a target rectangle by performing ankle dorsiflexion at 10 ± 0.5% MVC. Color of the cursor changed based on the β power in the MU pool. Subjects were asked to keep the cursor inside the force target and change the cursor color to either blue (down-modulation of the β activity) or red (up-modulation of the β activity). In a control condition, no feedback on the β feature was provided and, instead, the cursor turned white when placed inside the target.

The experimental paradigm for both experiments is visualized in Figure 1*A*.

#### Preexperimental processing

Before the start of the experiments, subjects were asked to perform a single maximum dorsiflexion of the ankle to estimate the maximum voluntary contraction level (MVC). The obtained MVC was set as a reference for the following experiment to ensure that stable forces were produced by the tibialis anterior muscle.

In addition to force feedback, experiment 2 also informed the subjects about the amount of β activity in the MU innervating the tibialis anterior muscle. For this, an online decomposition algorithm was used to decode MU activity in real time (Barsakcioglu et al., 2021). In order to estimate the separation matrix used to decode MU activity from the HDsEMG recordings, subjects were instructed to perform an additional ramp and hold task. This involved a 4-s period of linear increase in the contraction level departing from a relaxed position and reaching a contraction level of 10% of the MVC (ramp phase) and steady contraction at 10% of the MVC level held for 40 s (hold phase). The decomposed MU discharge behavior was visually inspected following established guidelines (Del Vecchio et al., 2020) while subjects were instructed to gradually increase the force because of dorsiflexion tup to 10% MVC to recruit MUs.

#### Experiment 1, force task

Experiment 1 aimed to assess the characteristics of cortical and MU β activity during constant isometric contraction at a mild force level. This experiment consisted of two blocks. In each block, subjects were provided with visually guided feedback on the exerted force and asked to follow a ramp and hold trajectory for 40 s at 10% MVC presented on a screen while EEG was recorded concurrently. Between blocks, subjects were instructed to rest to avoid muscle fatigue.

#### Experiment 2, β modulation

In experiment 2, the relationship between cortical and MU β was assessed while subjects were allowed control over MU β. For this, subjects were instructed to move a cursor inside a target rectangle by exerting a force because of ankle dorsiflexion at 10% MVC. While holding the cursor inside the rectangle, i.e., exerting a constant force at 10% MVC, subjects were asked to change the color of the cursor to match a presented target by modulating the MU β power at ∼20 Hz. Similar to experiment 1, EEG was recorded throughout experiment 2.

Experiment 2 consisted of three parts: (1) an initialization phase to determine all parameters necessary for real-time neurofeedback on the MU β activity; (2) familiarization phase to allow subjects to get familiar with the experimental neurofeedback environment and task; and (3) the neurofeedback task in which subjects were exposed to real-time feedback on the exerted force and MU β activity.

##### Initialization phase

The initialization phase mimicked the paradigm previously performed previously (Bräcklein et al., 2021). Subjects were asked to exert a force at 10% MVC for 40 s guided visually by a force trajectory. During this period, the underlying MU activity was used to identify the most prominent peak inside the β band of the intramuscular coherence (IMC). The IMC was used in this case as it allowed us to estimate the common input to the MU pool at a given frequency (Castronovo et al., 2015; Dideriksen et al., 2018). The power inside a 5-Hz band of the cumulative MU spike train (CST) centered around the IMC peak in the β band was extracted online using a third-order Butterworth filter. The mean of this β feature in the initial training block was used for normalization during the neurofeedback part in experiment 2. The logarithm of this normalized β feature was then fitted to a Gaussian distribution to provide feedback on the β activity using a color code. Specifically, a blue-to-white-to-red colormap was mapped to the logarithmical β feature ranging from 2 SDs below the mean (blue) to 2 SDs above the mean (red), while the mean was coded via the color white (see Fig. 1*B*). If the β feature value was outside the range of the colormap, i.e., more than 2 SDs off the mean, the displayed color was set to the closest extrema (either blue or red).

##### Familiarization phase

The familiarization phase provided subjects with the same feedback environment as they experienced later in the neurofeedback task. Subjects were instructed to move a cursor up into a target rectangle by modulating the force exerted during dorsiflexion of the ankle. This target rectangle was centered at 10% MVC with a lower and upper bound at 9.5% and 10.5% MVC, respectively. The cursor's color changed accordingly to the underlying β feature and its corresponding value in the blue-white-red colourmap. If the cursor was outside the target rectangle, its color was changed to black. Hence, subjects only received feedback on the underlying β feature when the cursor was inside the target. By doing this, subjects were encouraged to exert stable forces. Cursor position and color were updated every 125 ms. The β feature amplitude was averaged across the amplitudes observed in the seven most recent 125-ms buffers analyzed as previously performed by Bräcklein et al. (2021). Subjects had ∼10 min to get themselves familiar with this neurofeedback environment.

##### Neurofeedback task

The neurofeedback task was divided into multiple blocks. Subjects were asked to perform a minimum of three and a maximum of six blocks of training before three last consecutive blocks were used for further analysis. Each block consisted of three trials. Each trial started with subjects contracting their tibialis anterior muscle to produce ankle dorsiflexion forces that moved the cursor inside the target rectangle at 10% of the MVC. Once the cursor was within the target rectangle, the force produced had to be kept constant for 30 s while β activity had to be modulated. Specifically, subjects were asked to either keep the cursor blue for as long as possible (β down-modulation condition), or red (up-modulation condition). In a third condition, no feedback on the underlying β activity was given (the cursor stayed white when held inside the target; see Fig. 1*B*). The color target indicating the modulation condition of each trial, was provided verbally by the experimental instructor and as visual clues by the color of the cursor edge. Hence, the cursor edge was blue when subjects were asked to keep the cursor blue (down-modulating MU β), red (up-modulating β), or black if no neurofeedback on MU β was provided. Per block, each modulation condition was presented once in a randomized order. Between each trial, subjects rested for at least 1 min to minimize muscle fatigue.

### Analysis

#### Spectral analysis

The time-frequency representation of the CST and the surface Laplacian EEG was obtained using the continuous wavelet transform implemented via the cwt function in MATLAB (version 2018b, MathWorks Inc.). The cortico-muscular coherence (CMC) was estimated using magnitude-squared wavelet coherence implemented via the MATLAB function wcoherence. A similar approach was chosen to estimate the temporal evolution of the IMC via a custom MATLAB script built on the wcoherence function. To estimate the IMC, the MU pool was split into two randomly selected subpools of equal size. The magnitude-squared wavelet coherence between the CSTs of both MU subpools was calculated. This step was repeated over 100 iterations, always choosing a different configuration of MU subpools. The IMC was obtained by averaging the coherence estimates obtained during the 100 iterations.

**Figure 2.**
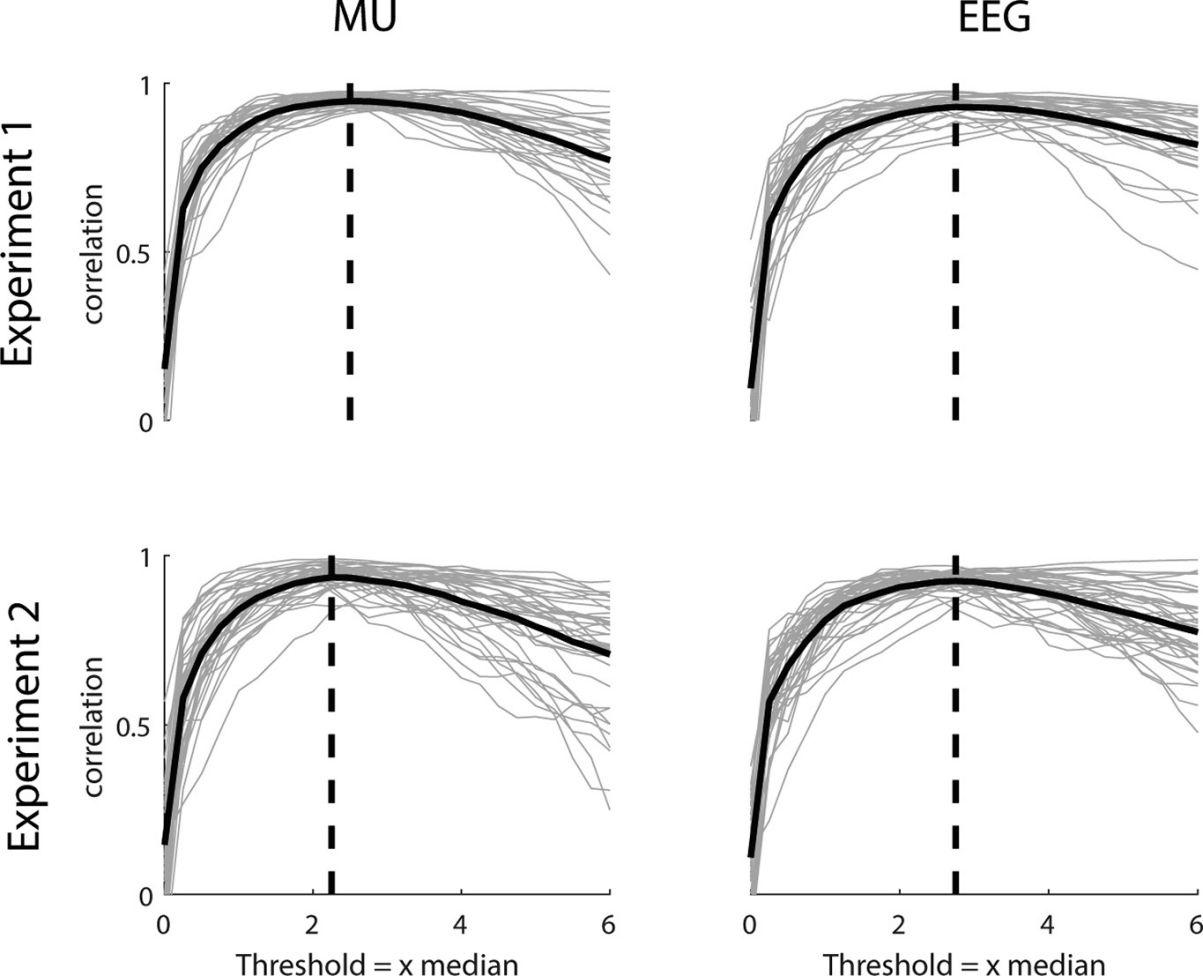
β Burst threshold estimation. Correlation between β band power and number of samples above threshold for experiment 1 (top) and experiment 2 (bottom) for the MU (left) and EEG (right) data. Gray lines indicate single blocks while solid black lines indicate mean across blocks. For experiment 2, only the control condition was used. Dashed black lines indicate maximum correlation value and corresponding threshold.

The β bursting activity present in the CST and EEG signals was extracted using a bandpass filter (13–30 Hz, fourth-order Butterworth). The envelopes of the bandpass-filtered signals were used to determine when β bursts occurred. The threshold above which the envelope was classified as a bursting event was empirically determined similar to the methods used previously (Shin et al., 2017; Little et al., 2019). For experiment 1, the envelopes from EEG and CST in each block were split into 1-s windows. In each window, the correlation between the power of the signal and the percentage of signal above the threshold was determined using the Pearson correlation coefficient and averaged across blocks. Hereby, the threshold was increased from 0 to 6 times the median in 0.25 steps. The threshold that resulted in the maximum correlation between power and percentage of signal above threshold was used to identify β events. This procedure was repeated for experiment 2 on block-level for the non-β power feedback trials. The results are visualized in Figure 2. For experiment 1, the empirically determined threshold was 2.50 and 2.75 times the median for CST and EEG, respectively. For experiment 2, it was 2.25 and 2.75 times the median for CST and EEG. Consecutive periods where the envelopes were above the threshold were marked as ON periods (β bursting events), similarly as previously performed (Echeverria-Altuna et al., 2021). Hence, the length of ON periods was used to estimate the duration of β events. The β event power was calculated as sum of all ON events divided by the recording time. The remaining periods, i.e., when the envelope was below the threshold, were identified as OFF periods. The time points of ON and OFF events were set to the center of the respective periods. To analyze neural activity around ON and OFF periods, the wavelet transposed spectra of CST and EEG, the wavelet CMC and IMC were averaged in 500-ms windows centered at the times of ON and OFF events. Furthermore, the percental mismatch between ON and OFF events was calculated as: ((ON – OFF)/OFF) * 100.

#### Experiment 1, force task

The HDsEMG recorded during 40 s of isometric ankle dorsiflexion at 10% MVC was offline decomposed into the underlying MU activity using the algorithm proposed previously (Negro et al., 2016). The decomposition results were manually inspected as detailed previously (Del Vecchio et al., 2020). To control if the identified bursts in the EEG and the MU pool result from underlying amplitude modulations or in contrast from isolated bursting events, the lagged coherence method was employed (Fransen et al., 2015) using the NeuroDSP Python toolbox (Cole et al., 2019). This spectral measure examines coherence between the signal and a delayed version of the same signal at each frequency. If the lagged coherence is large, it provides evidence that the observed bursting events occur in periodically and thus may be because of an underlying modulation. However, when the examined signal occurs in de-coupled events, detached from any ongoing modulations, the lagged coherence is smaller. The power spectral density was calculated using Welch's method (2-s window, 50% overlap) and normalized between 1 and 40 Hz.

#### Experiment 2, β modulation

The online decomposed MU activity was *post hoc* cleaned from artefacts. Action potentials that were fired with an instantaneous discharge rate above 30 spikes per second (sps) were neglected. Only the 30-s time interval during which subjects were instructed to modulate the β activity while keeping the force constant were analyzed. In addition, the β activity and discharge rate were recalculated by neglecting MUs that had an average discharge rate below 5 sps or above 30 sps or a discharge rate coefficient of variation (CoV) above 0.5 in any of the recorded blocks. The resulting cleaned pools of MUs were used in the subsequent analysis, also for example, to recalculate the β feature and wavelet transformed CST activity, CMC, and IMC.

Functional values obtained during up-modulation and down-modulation of MU β activity, such as the mean force, β amplitude, average rectified EMG, i.e., global EMG, bipolar EMG, and the corresponding CoVs to all values mentioned before, and the mean MU discharge rates were normalized by the averaged values obtained during the control condition (when no neurofeedback on the MU β activity was provided). The wavelet transformed CST and EEG, CMC, and IMC were interpolated to transform the logarithmical frequency scale into a linear one for further analysis to ensure an equally weighted representation of all frequencies. The results were averaged inside the entire β band (13–30 Hz) and within in 500-ms window centered around the ON-triggered averaged. The values obtained during neurofeedback were normalized by the corresponding values obtained during the control condition.

The custom scripts used for analysis are available on reasonable request from the corresponding author.

### Statistics

Statistical analysis was performed via SPSS (IBM) and custom MATLAB routines. Results were reported as mean ± SD. Significant clusters of β activity in the difference in the time-frequency representation of β ON and OFF events were determined using the cluster-level analysis proposed by Maris and Oostenveld (2007). In brief, this approach assessed clusters of adjacent samples in both frequency and time dimensions under a single permutation distribution (we used 10,000 permutations and an univariant clustering threshold of 0.05). This approach allows to bypass multicomparison issues present in multidimensional data. The characteristics of β bursting events in the MUs and the EEG were compared by using two-sided paired *t* tests. The effect of volitional β modulation on multiple motor behavioral properties of the innervated leg were tested by a repeated measures MANOVA. Hereby, the independent variables were the different modulation conditions, i.e., β down-modulation and up-modulation. Dependent variables were the mean force, mean rectified EMGs of agonist and antagonist muscles, the CoV of these values and the mean discharge rates of the decomposed MUs across subjects. Differences in the mean β feature amplitude were assessed by two-sided paired *t* tests. To assess whether the temporal evolution of the modulated β feature correlated with muscle activation, the correlation coefficient between the exerted force, the rectified EMG of the agonist muscle or the discharge rate of the identified MU pool, and the β feature were estimated using the Pearson correlation coefficient. To do this, force, rectified EMG and discharge rate were postprocessed in a similar fashion as the β feature, i.e., corresponding values were averaged per each recording buffer.

The difference in β event features at the cortical and MU level was assessed using linear mixed models. Linear mixed models were also used to evaluate the effect of volitional β modulation at MU level on the β bursting characteristics and spectral values, such as wavelet-transformed CST and EEG, CMC, and IMC, on single blocks, in which the difference between β down-modulation and up-modulation was the dependent variable and the subject-wise grouping a random effect. Values during up-modulation and down-modulation were normalized using data from the nonfeedback condition as described in 2.4.3. The partial η^2^ (η_p_^2^) was used to assess the effect size of the changes between β modulations. Values >0.14 indicate that a “large” effect can be observed in the particular comparison (Cohen, 1988). The threshold for statistical significance was set to *p* < 0.05.

## Results

### Experiment 1, force task

**Figure 3.**
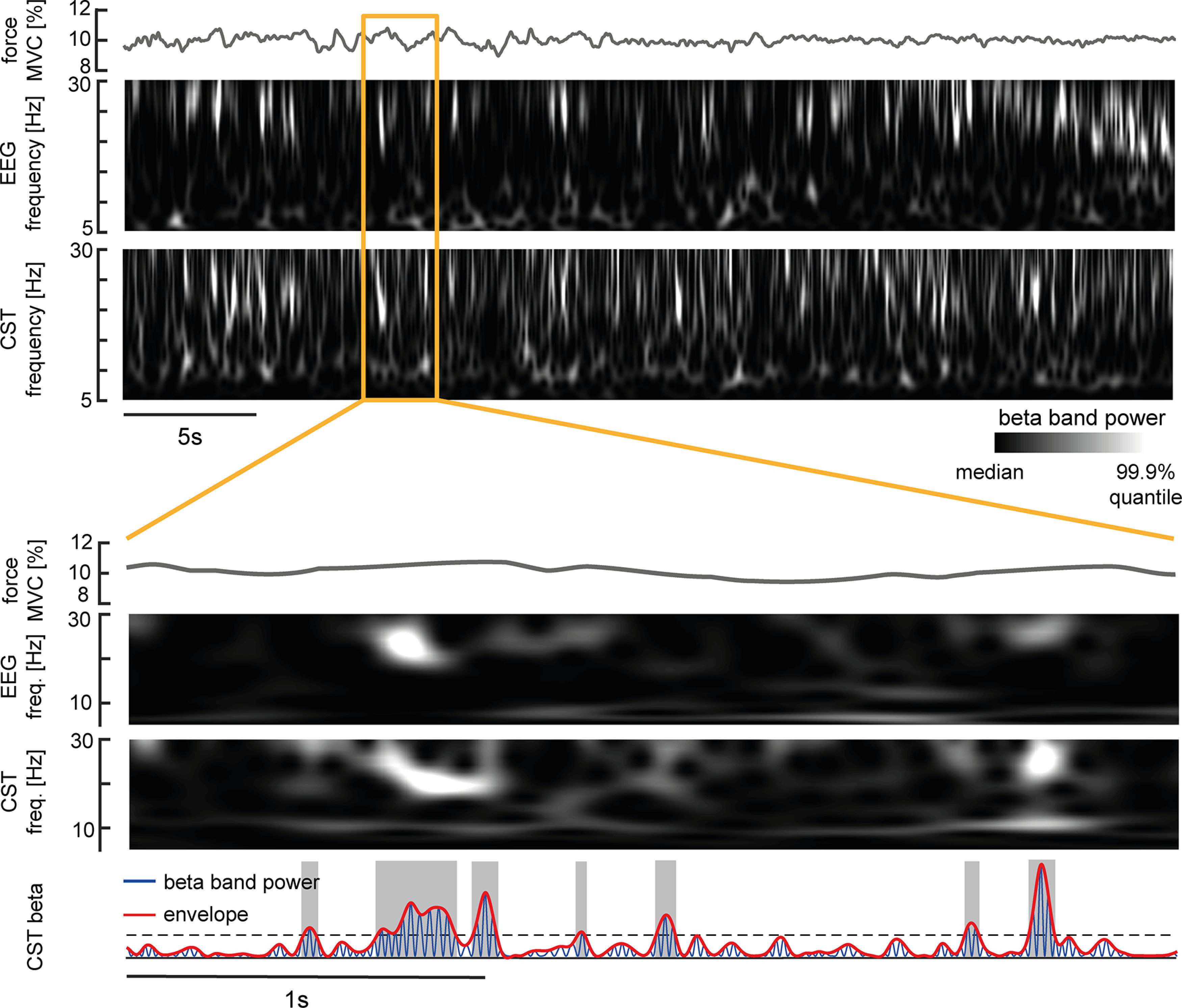
β Power present in the EEG and MU pool shown in a representative subject. Top, Force because of dorsiflexion of the ankle, interpolated time-frequency spectrum inside the β band for surface Laplacian EEG and CST via continuous wavelet transform. Bottom, Zoom-into force, interpolated time-frequency-spectra of surface Laplacian EEG and CST, and β band power (blue) and maxima envelope (red) extracted from the bandpass filtered CST. The black dashed line indicates the threshold used to identify β bursts (ON, gray shaded areas) and valleys in between bursts (OFF).

In total, 22.73 ± 7.95 MUs per block were identified in experiment 1. Figure 3 visualizes the time-frequency spectra inside the β band of cortical (EEG signals) and muscle (the CST generated with the decomposed MUs) signals during a period of isometric ankle dorsiflexion at 10% MVC. Both spectra indicated that β activity at the cortical and muscle levels occurred in short intervals, i.e., bursts of activity, while subjects held constant forces. The zoomed-in plot (Fig. 3, bottom) suggested that some bursts observed in the muscle overlapped with bursts observed in the EEG.

**Figure 4.**
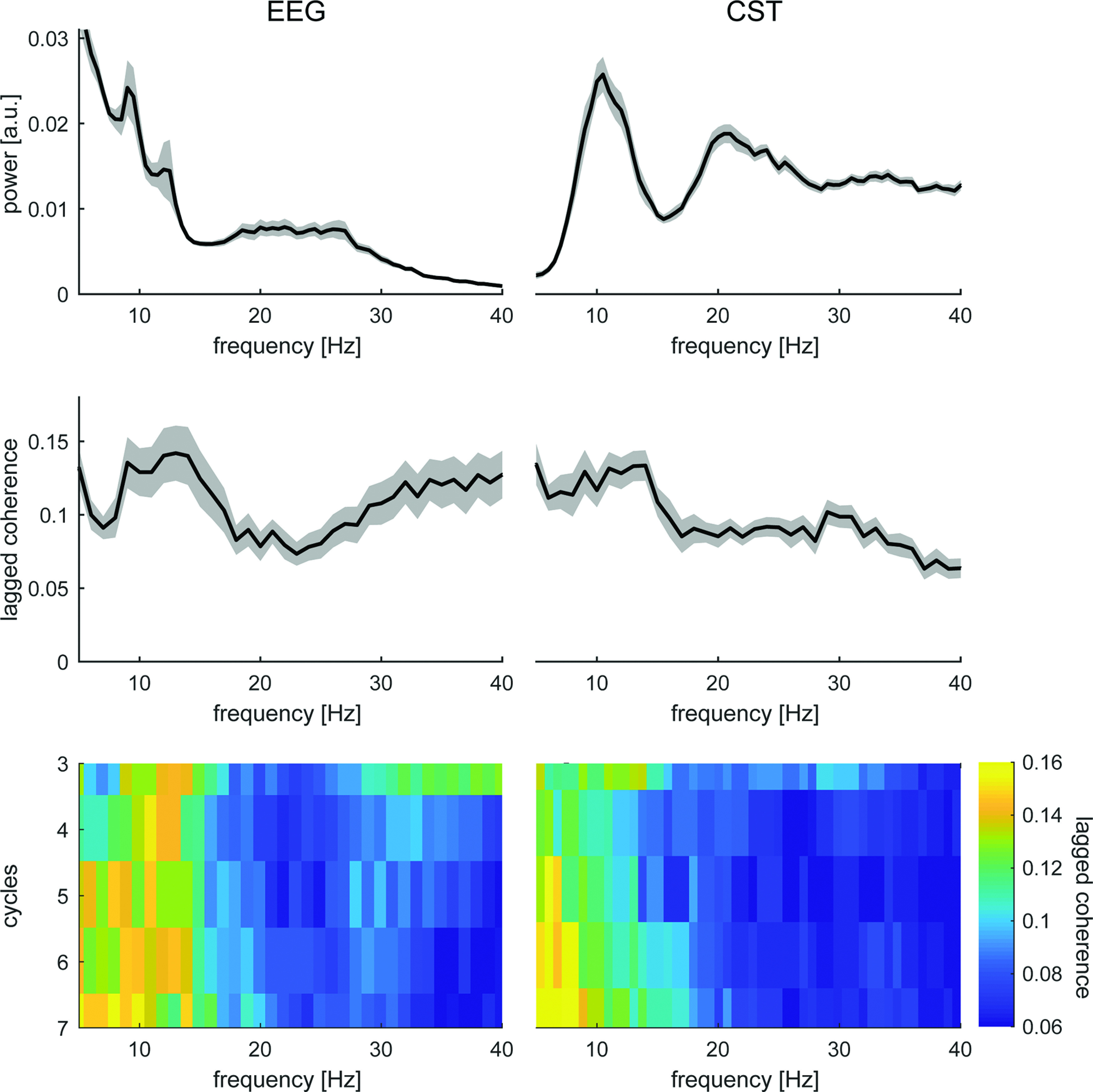
Lagged coherence analysis for EEG (left) and MU activity (right). Top, Mean power spectral density normalized between 1 and 40 Hz across all blocks. Shaded areas indicate standard error of the mean (SEM). Middle, Mean lagged coherence at three cycles across all blocks. Shaded areas indicate SEM. Bottom, Mean lagged coherence for cycles 3–7 across blocks.

While the observed β burst might occur as infrequent uncoupled bursting events, they could also result from an underlying amplitude-modulated oscillation. Hence, we conducted a control analysis to assess whether β bursts in MUs and EEG result from a sustained amplitude modulation. In this case, the phase inside the β band should predict the phase in upcoming cycles. In contrast, if these bursts do not originate from an underlying sustained modulation, the current phase inside β should correlate less with future cycles (Fransen et al., 2015). Figure 4 illustrates that although both cortical and MU show prominent β activity in their spectra, the lagged coherence decreases inside this range compared with other spectral components. Further, this effect seems prolonged across multiple cycles. This indicates that in both EEG and MU activity, β bursting events seem to be isolated, thus not resulting from underlying modulation.

**Figure 5.**
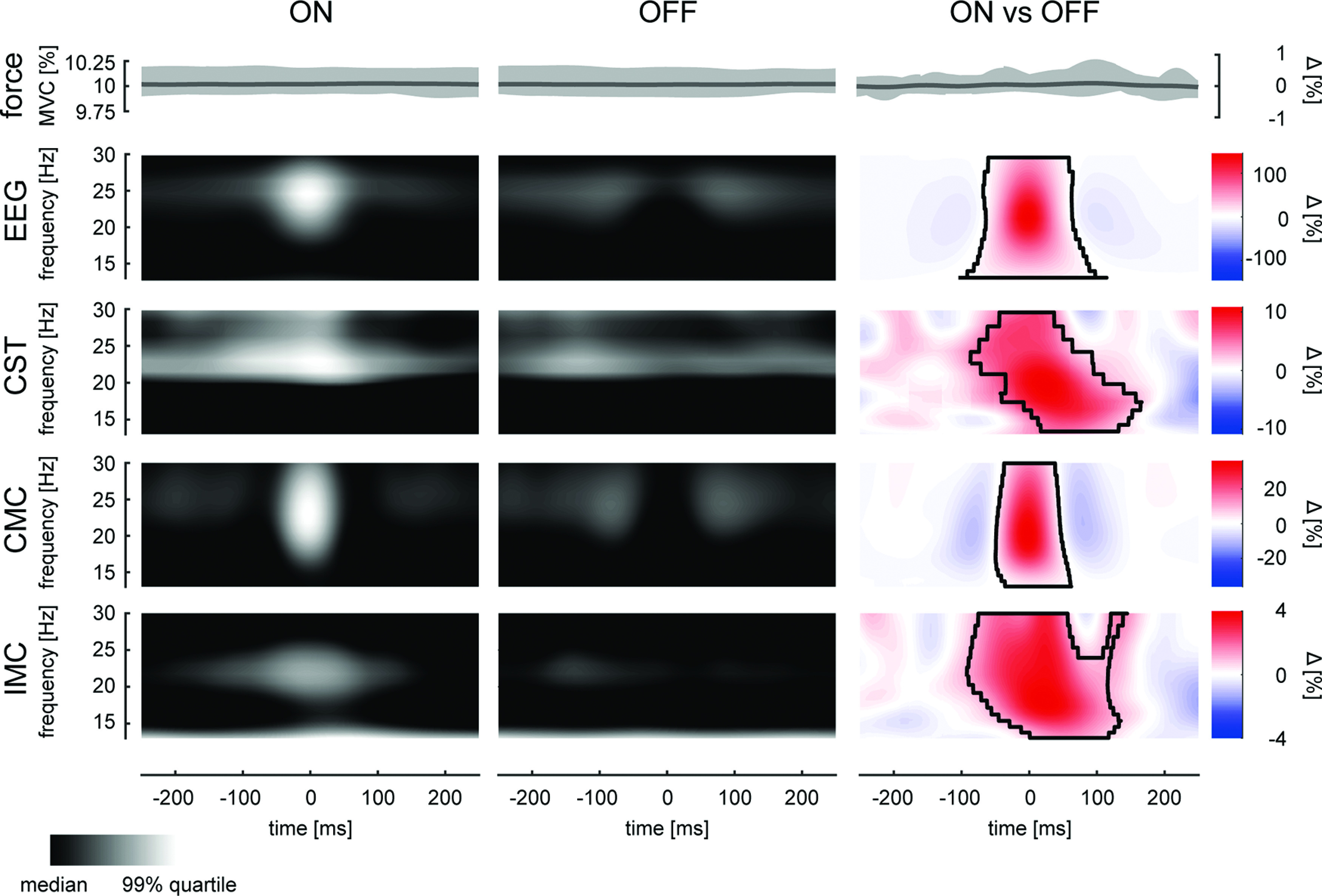
Neural activity during β bursting events present in the EEG. ON and OFF periods were aligned and averaged across blocks. From top row to bottom, Force (shading indicates 95th percentile), interpolated wavelet-transformed EEG, wavelet-transformed-MU activity, CMC, and IMC, at the center time points of ON periods (left), OFF periods (center), and percental mismatch (right). Black boundaries indicate significant clusters (*p* < 0.05).

To understand activity around the short-lived β bursts found in the EEG and CST signals, the wavelet-transformed data were averaged at the center of ON and OFF periods found in the EEG across blocks. Figure 5 visualizes these triggered averages for the wavelet-transformed EEG, CST, the CST-EEG coherence (CMC), the IMC, and the force profile at respective time intervals. While the exerted force did not significantly change between ON and OFF periods (no significant clusters, always *p* > 0.05), β activity present in the EEG was significantly pronounced during ON relative to OFF periods in a cluster at the center of EEG β events (*p* = 0.024). Also, β activity in the CST was pronounced during ON periods compared with OFF, despite the time points of ON and OFF being determined by the EEG activity (*p* = 0.042). It is worth noting that the maximum difference between ON and OFF in the EEG was around time lag 0 (–0.49 ms), while the maximum difference in the CST was delayed by 24.41 ms. Furthermore, a significantly pronounced bursting activity in the CMC was observed (*p* = 0.001). Similarly, the results suggested that the IMC was also of transient behavior inside the β band (IMC, *p* = 0.026).

**Figure 6.**
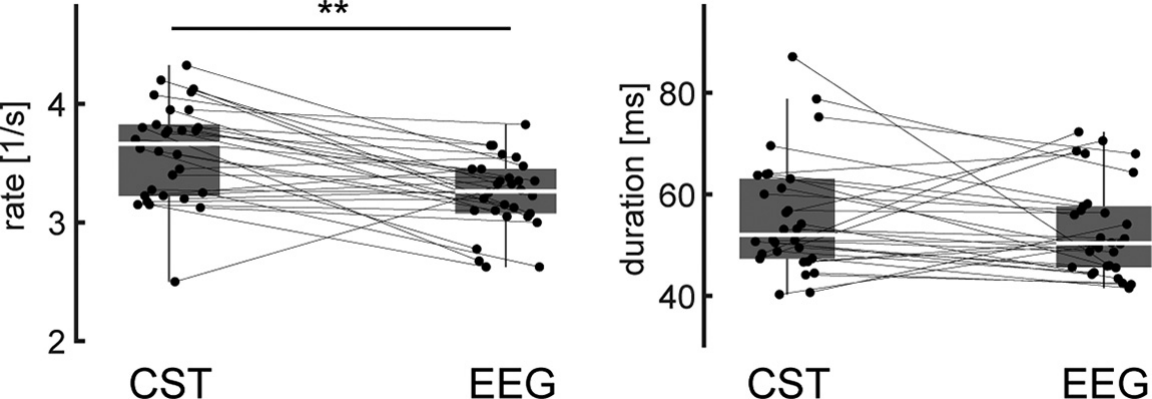
Relationship between β bursts observed at the cortical and muscle levels. The rate at which β events occurred (left) and their mean duration (right) are shown for cortical (EEG) and peripheral (CST) signals across blocks by their median and quantiles. Values for individual blocks are marked in gray and connected observation sides of β events (i.e., CST and EEG). **p < 0.01.

The previous results indicated that β activity observed in cortical and muscle recordings occurred in bursts. Moreover, the significant β activity in the CST identified during EEG β bursting events suggested that β bursts in the MU overlapped with those present at the cortical level. This confirms previous observations made using surface EMG signals (Echeverria-Altuna et al., 2021). In addition, we observed that the common input inside the β range to the MU pool was of bursting behavior and appeared to be time-locked to cortical β bursts. To further assess how β bursts observed in the MU pool matched with the β bursts in the EEG we compared the rate and duration of the β bursts extracted from the CST and EEG (Fig. 6). β Events observed at the MU level appeared at a rate of 3.56 ± 0.41 events per second while β events in EEG at a slightly but significantly lower rate of 3.23 ± 0.30 (*p* = 0.003, η_p_^2^ = 0.469). There was no significant difference detected between the average duration of the β bursts observed on the MU level (55.61 ± 11.27 ms) and the bursts in the EEG (53.10 ± 9.23; *p* = 0.398, η_p_^2^ = 0.052).

### Experiment 2, β modulation

Results from experiment 1 showed that β activity occurs in bursts both at the cortical and muscle levels. Moreover, the bursts observed at both levels are similar in features such as duration and rate of events and appear to be temporarily aligned with a small offset. These results therefore support the notion that β activity in the EEG and CST have a shared underlying source. If this is the case, it is expected that modulation of β activity at the MU level should correspond to a similar modulation of cortical β observed in the EEG. To test this, experiment 2 used a novel neural interface based on real-time decomposition of MU activity from the interference EMG.

**Figure 7.**
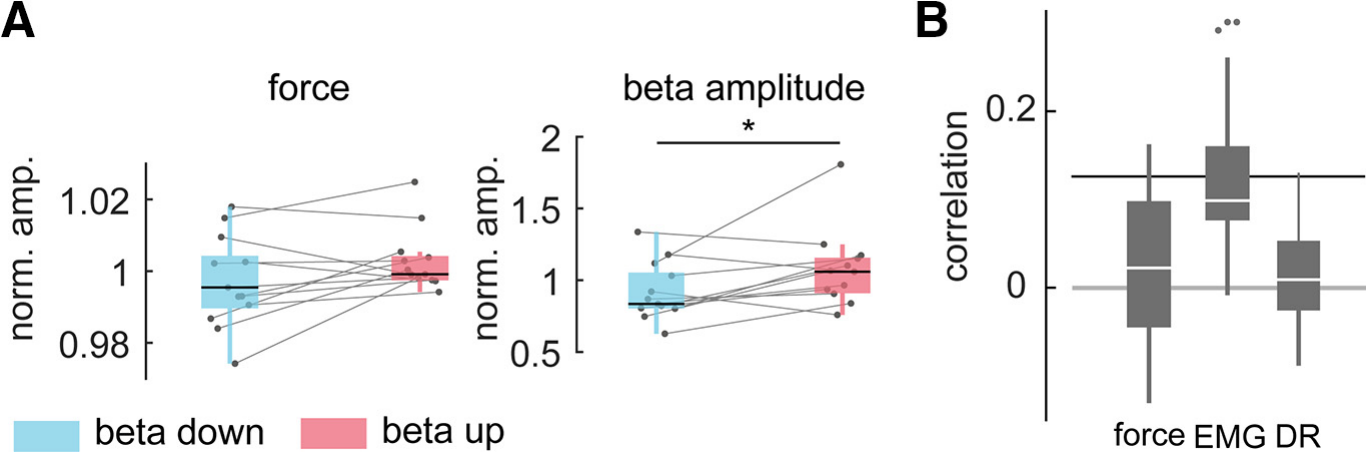
Functional values during β power modulation. ***A***, Mean force and β feature amplitude (normalized by mean amplitude during nonfeedback condition) during down-modulation and up-modulation conditions (blue and red, respectively) shown by their median and quantiles all subjects. Gray points indicate the mean value per subject, while gray lines combine data of the same subject. *p < 0.05. ***B***, Temporal correlation between the β power feature and the force, global EMG of the tibialis anterior and the mean discharge rate (DR) shown across subjects with their median and quantiles. Black bar indicates significance level of correlation. (norm. amp.: normalized amplitude).

In this online experiment 11.92 ± 2.48 MUs per subject were identified and tracked in real time. Subjects could significantly reduce the normalized mean β amplitude during down-modulation to 0.91 ± 0.20, compared with up-modulation at 1.07 ± 0.26 (two-sided paired *t* test, *t*_(12)_ = −2.454, *p* = 0.030; see Fig. 7*A*). In the context of volitional MU β modulation, neither the mean exerted force nor other functional measures of the innervated leg changed significantly (repeated measures MANOVA Wilks' λ corrected, *p* = 0.424, η_p_^2^ = 0.811). Furthermore, across all subjects, no temporal correlation between the β feature and the force, rectified EMG of the tibialis anterior muscle, and discharge rate of MUs were detected (Fig. 7*B*; all medians are below the significance level). Taken together, these results suggested that subjects were able to modulate the β band activity present in a MU pool without critically altering the motor output.

**Figure 8.**
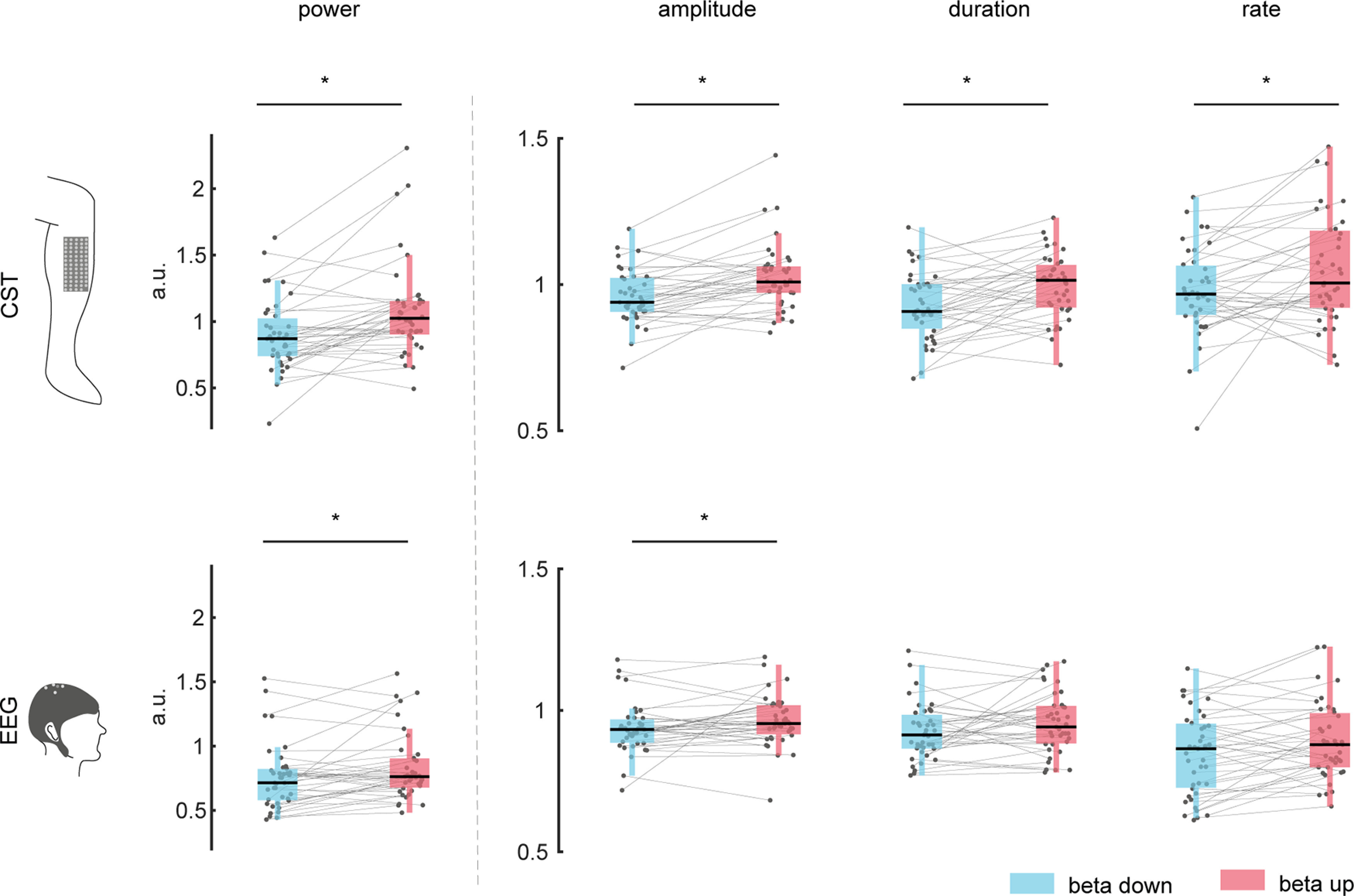
Normalized β events features during modulation. Mean power, amplitude, duration, and rates of β events are shown across blocks. Corresponding values for β down-modulation (blue), and up-modulation (red) are normalized by the control condition (no neurofeedback on β activity). The top row shows values observed on the MU level (CST) and the bottom one for EEG level. Gray dots indicate values for single blocks. Gray lines combine values corresponding to the same block. *p < 0.05. (a.u.: arbitrary units). (a.u.: arbitrary units).

To study the impact that modulation of β activity in the MU pool has on cortical β activity, we compared the burst power and the three burst features that contribute to the power estimate, i.e., peak amplitudes of the β bursts, the bursts durations, and the number of bursts, between β down-modulation and up-modulation conditions normalized by the corresponding values obtained when no β feedback was provided (Fig. 8). The power of the β bursts in both CST and EEG increased during up-modulation compared with down-modulation from 0.89 ± 0.27 to 1.09 ± 0.37 (*p* = 0.003, η_p_^2^ = 0.540) in the CST, and from 0.75 ± 0.25 to 0.83 ± 0.26 (*p* = 0.013, η_p_^2^ = 0.415) in the EEG. The amplitudes of β bursts in the CST and in the EEG were significantly higher in the up-regulation condition than in the down-modulation condition (CST: from 0.96 ± 0.09 to 1.02 ± 0.12, *p* = 0.002, η_p_^2^ = 0.581; EEG: from 0.94 ± 0.09 to 0.96 ± 0.09, *p* = 0.038, η_p_^2^ = 0.311). The duration of the β events did also change between conditions at the MU level from 0.93 ± 0.12 to 1.00 ± 0.11 during down-modulation and up-modulation, respectively (*p* < 0.001, η_p_^2^ = 0.652) but was not significant at the cortical level with longer durations of β events during up-modulation (from 0.92 ± 0.10 to 0.96 ± 0.11, *p* = 0.079, η_p_^2^ = 0.235). The rate of observed β events at the MU level increased significantly from 0.98 ± 0.15 to 1.05 ± 0.18 (*p* = 0.023, η_p_^2^ = 0.363). On average, the rate of β events did also increase at the cortical from 0.85 ± 0.14 to 0.89 ± 0.14, but this effect was marginally not significant (*p* = 0.058, η_p_^2^ = 0.268).

**Figure 9.**
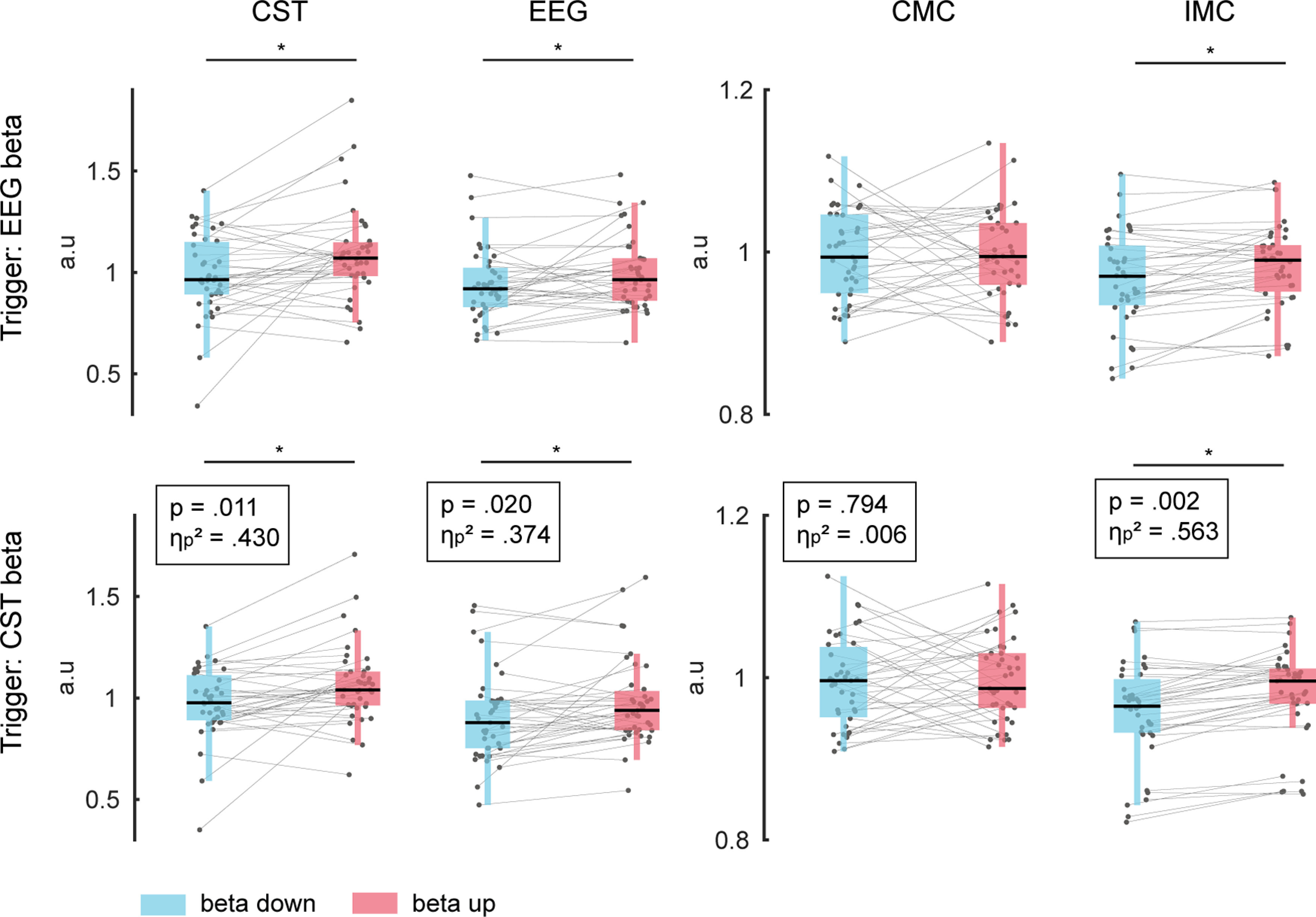
Impact of volitional β feature modulation on spectral measures. From left to right, β band power extracted from the MU activity and EEG, β band coherence in the CMC and IMC across subjects during β feature down-modulation (blue) and up-modulation (red). Mean values were extracted from a 500-ms window centered around the ON periods identified in the EEG (top) and MU activity (CST, bottom) and were normalized by the corresponding values obtained during the control condition (no β neurofeedback). Gray dots indicate values for single block, while gray lines combine values corresponding to the same block. *p < 0.05. (a.u.: arbitrary units).

The appearance of β bursts in the EEG and MU activity changed during volitional β feature modulation. Figure 9 shows the impact of volitional β modulation on the MU and EEG β activity during β ON events. The spectral power in the β band during ON events increased significantly in the CST from 0.99 ± 0.20 to 1.09 ± 0.23 (*p* = 0.019, η_p_^2^ = 0.378) and in the EEG from 0.90 ± 0.11 to 0.94 ± 0.11 (*p* = 0.026, η_p_^2^ = 0.351) during down-modulation and up-modulation conditions, respectively. Similarly, the IMC increased significantly during up-modulation from 0.97 ± 0.06 to 0.98 ± 0.05 (*p* = 0.034, η_p_^2^ = 0.321) suggesting a stronger common input in the β band during the up-regulation condition. Interestingly, the CMC did not change significantly from 1.00 ± 0.06 to 1.00 ± 0.06 between conditions (*p* = 0.994, η_p_^2^ = 0.000), which implies that while the common input to the MU inside the β range increased during β up-modulation relative to down-modulation, the spectral connectivity between cortical β and MU β remained unaffected. These results indicated that cortical β power mirrored the changes in the MU. Finally, it should be noted that the same overall effects were observed when using β bursting events in the CST to define the timing of ON periods (see Fig. 9).

## Discussion

We studied the correspondence of cortical β activity with β oscillations found in the output of spinal motor neurons. To do this, we assessed how cortical and peripheral β bursting events relate to each other during muscle contractions. We then used a MU-driven neurofeedback approach to modulate the β inputs to muscles to test whether cortical β activity followed the modulation of peripheral β activity. Our results demonstrate, for the first time, that β activity present in a MU pool appears in isolated bursts that closely correspond to the β activity observed at the cortical level. In addition, when modulated at the periphery, cortical β showed the same modulation pattern. We conclude that β activity in the periphery is mainly determined by cortical projections.

The common β activity present in the MU population strongly corresponded to the cortical β projections. We showed that β activity present in a MU pool is short-lived and shares the characteristics of the cortical β rhythms, i.e., rate and duration of β events. Moreover, the common input to the MU pool inside the β range and the resulting MU β activity were time-locked and followed cortical β rhythms by tens of milliseconds. Although determining the transmission delay by only analyzing the β power is not robust against noise that may mask the underlying shape of β bursts, our observation is in strong agreement with previous investigations using the averaged CMC (Mima et al., 2000; Ibáñez et al., 2021). When we asked subjects to perform volitional modulations of the β activity present in the MUs via a novel neurofeedback paradigm (Bräcklein et al., 2021), changes in the cortical β power were shown to be coherent with those induced in the periphery. These findings suggest a strong and stable correspondence between peripheral and cortical β oscillations during steady force contractions.

Although the effective β activity at the MU level could potentially result from other neural centers (Thompson et al., 2019), as it was suggested to be the case for MU activity in the α range (8–12 Hz) during tremor (Christakos et al., 2006), it seems that these noncortical contributions may be minimized or suppressed in the context of cortical inputs during isometric contractions. If their contribution would have superseded the presence of cortical projections at the MU level, the resulting β activity in the periphery would be expected to differ from β patterns observed at the cortical level. Moreover, the common input to the MUs inside the β band was increased during volitional up-modulation of the MU β power while the connectivity between cortical and peripheral sites remained unaffected (Fig. 9). Hence, the coherence between the cortical regions and the MU pool inside the β band (CMC) did not change, but the strength of the common input received by the MU pool (IMC) did. This provides additional evidence for MU β signals mainly emerging from the cortical sites: if successful β modulation resulted from additional modulation of noncortical sources, the CMC would have been affected by the volitional β feature modulation (Negro and Farina, 2011).

The dominance of cortical β inputs to muscles contrasts with the observed lack of direct influence on the produced force. No significant relationship between the force output of the tibialis anterior muscle and the presence of β rhythms in the innervating MU pool was detected. Still, despite the absence of any evidence for a direct link between β bursts and the motor output, β oscillations at the MU level could determine a nonlinear effect on the neural drive to the innervated muscle and therefore on the force output (Watanabe and Kohn, 2015). Our results show, however, that these β events at the MU level are infrequent, i.e., approximately four events per second (Fig. 6). While a stationary β that changes amplitude continuously, as simulated previously (Watanabe and Kohn, 2015), may influence force control, a bursting β is very unlikely to do so since the corrections in force would be far too slow to improve steadiness. Alternatively, the motor system could use the observed β events as a sonar signal integrating sensory information from the muscle (Baker et al., 2006), yet this hypothesis requires further experimental validation. During experiment 2, when subjects were instructed to modulate MU β power, and cortical β changed coherently, the exerted force remained unchanged. This provides further evidence that apart from the timing of β bursting events, also the modulation of the β event amplitude does lie inside a motor null-space relative to force production. Hence, the strong link between cortical and spinal neurons via β activity observed in this study did not seem to have any direct influence on motor output.

When subjects were exposed to neurofeedback on the MU β activity, β modulations at the cortical and MU levels were mainly driven by altering the amplitude. Also, rate and duration of β events increased during β-up-modulation; however, this effect was only significant at MU level. It yet remains unknown what underlying mechanism led to a volitional increase in β power via increase in the amplitude of β bursts. One possible explanation would be that subjects were able to recruit larger cortical networks involved in the projection of β activity to the muscle. It was previously shown that the duration of β bursts was not affected by the performed motor task in normal conditions (Echeverria-Altuna et al., 2021). Here, we observed, although not always significant, slightly longer periods of β events during β up-modulation compared with down-modulation of MU β. Subjects did not receive feedback on the instantaneous amplitude of β events, nor about their duration or rate. Instead, the feedback provided on the β feature amplitude during experiment 2 was smoothed with a moving average and aimed to motivate subjects to modulate the β activity across the entire duration of the trial, i.e., suppressing or promoting β activity as long and as often as possible. Additional experiments with different neurofeedback approaches (e.g., using the instantaneous behavior of β events) are necessary to investigate whether subjects could learn to modulate other characteristics of β activity in the brain and the muscles. This would be highly useful to advance our understanding of the possible roles of β oscillations in movement.

Finally, the strong presence of cortical projections at the MU level opens up new means of studying cortical β: peripheral neural interfaces, such as presented previously (Barsakcioglu et al., 2021), would allow an indirect yet reliable window into cortical activity and may contribute to an advanced understanding of the functional role of β oscillations in the human motor nervous system by complementing traditional interfaces, such as based on EEG or magnetoencephalography. We showed that by closing the loop with a peripheral neural interface based on MU activity, subjects could volitionally modulate the power of cortical β bursts. This could provide new possibilities to exploit cortical β, for example, as a control signal for virtual or robotic effectors (Dominijanni et al., 2021; Eden et al., 2022).

In conclusion, we have shown for the first time that the final neural drive to muscles contains bursting β activity. Moreover, these β bursts in the MU behavior shared the appearance and were time-locked to those observed on the cortical level. Volitional modulation of MU β activity was accompanied by coherent changes in cortical β manifesting the strong correspondence between cortical and MU β. The observed bursting activity inside the β band appeared in infrequent events at low rate and thus may, at most, influence force generation as a disturbing factor rather than supporting accurate force control. Cortical β oscillations seem to be the main contribution to MU β activity and the strong correspondence between cortical and peripheral β suggests the potential use of peripheral neural interfaces to track and modulate cortical activity.
